# Analysis of Attentional Bias towards Attractive and Unattractive Body Regions among Overweight Males and Females: An Eye-Movement Study

**DOI:** 10.1371/journal.pone.0140813

**Published:** 2015-10-19

**Authors:** Petra Warschburger, Claudia Calvano, Eike M. Richter, Ralf Engbert

**Affiliations:** 1 Department of Psychology, Division of Counseling Psychology, University of Potsdam, Potsdam, Germany; 2 Department of Psychology, Division of Experimental and Biological Psychology, University of Potsdam, Potsdam, Germany; University of Verona, ITALY

## Abstract

**Background:**

Body image distortion is highly prevalent among overweight individuals. Whilst there is evidence that body-dissatisfied women and those suffering from disordered eating show a negative attentional bias towards their own unattractive body parts and others’ attractive body parts, little is known about visual attention patterns in the area of obesity and with respect to males. Since eating disorders and obesity share common features in terms of distorted body image and body dissatisfaction, the aim of this study was to examine whether overweight men and women show a similar attentional bias.

**Methods/Design:**

We analyzed eye movements in 30 overweight individuals (18 females) and 28 normal-weight individuals (16 females) with respect to the participants’ own pictures as well as gender- and BMI-matched control pictures (front and back view). Additionally, we assessed body image and disordered eating using validated questionnaires.

**Discussion:**

The overweight sample rated their own body as less attractive and showed a more disturbed body image. Contrary to our assumptions, they focused significantly longer on attractive compared to unattractive regions of both their own and the control body. For one’s own body, this was more pronounced for women. A higher weight status and more frequent body checking predicted attentional bias towards attractive body parts. We found that overweight adults exhibit an unexpected and stable pattern of selective attention, with a distinctive focus on their own attractive body regions despite higher levels of body dissatisfaction. This positive attentional bias may either be an indicator of a more pronounced pattern of attentional avoidance or a self-enhancing strategy. Further research is warranted to clarify these results.

## Introduction

Being overweight or obese is highly stigmatizing in nearly all areas of everyday living [1,2]. There is abundant literature suggesting that these negative experiences contribute to the impaired psychosocial health and increased body dissatisfaction reported by overweight and obese individuals [3,4]. A disturbed body image can manifest itself in “…affective, cognitive, perceptual, or behavioral disturbance that is directly concerned with an aspect of physical appearance” [5]. A negative body image is highly prevalent in overweight and obese individuals [6,7], and is strongly associated with cognitive-affective and behavioral elements such as body dissatisfaction, body-related avoidance, and body checking behavior. A higher frequency of body checking (e. g., repeatedly checking the appearance of “critical” body regions) and avoidance (e.g., not wearing a swimsuit, wearing very loose clothing) has been described in overweight and obese individuals (e.g., [8,9]), especially when obesity is associated with binge eating [10,11]. Gender differences are consistently found in the literature, with women reporting higher weight and shape dissatisfaction (e. g., [8,12]) and greater idealization of a thinner body shape [6] than men. Such a negative evaluation of one’s own body may trigger and exacerbate the development of a disturbed body image.

Attentional biases towards distinct body regions might add to the vicious, self-perpetuating cycle. Literature with respect to eating disorders suggests an attentional bias towards body- and food-related information [13,14], which plays an important role in the development and maintenance of these disorders. The assessment of eye movements is considered an objective, direct and non-invasive tool for examining attention allocation and attentional biases by providing information on fixation duration and number of fixations over certain time spans. To date, only a small number of studies have applied this approach using different samples and methodologies, making it difficult to draw conclusions.

In one of the first such studies, Freeman et al. [15] observed that eating-disordered females focus more extensively on their own unattractive body parts compared to healthy controls. However, since the authors only included the subjects’ own pictures, it remains unclear whether individuals with high body dissatisfaction may focus on others’ unattractive regions as well. Such a relationship is supported by a recent study by Lykins, Ferris, and Graham [16], who found a positive association between the evaluation of one’s own body and attention allocation when viewing unfamiliar bodies.

In an elegant experiment, Jansen, Nederkoorn, and Mulkens [17] extended this research by combining the subjects’ own and control pictures in a group of eating-symptomatic students and normal controls. While the control group spent a comparable amount of time looking at their own most attractive and least attractive body parts, the eating-symptomatic students spent significantly more time looking at their least attractive body parts. When viewing the control pictures, a reverse pattern was found: The eating-symptomatic students fixated longest on the most attractive body parts (self-depleting bias / negative attentional bias), whereas the healthy controls focused longest on the least attractive ones (self-serving bias / positive attentional bias). A similar pattern of attentional bias was reported by Svaldi, Caffier, and Tuschen-Caffier [18] for females with binge eating disorder (BED) and by Blechert, Nickert, Caffier, and Tuschen-Caffier [19] for patients with bulimia nervosa (BN). Contrary to the above-mentioned results, two recent studies [20,21] did not observe relevant differences between high and low body dissatisfied or eating-disturbed individuals. Since obesity and eating disorders share common features, such as a high level of body dissatisfaction [7] and disordered eating patterns [22], one might also expect to find similar attentional patterns in obese individuals. However, to the best of our knowledge, only three studies have included an overweight sample in investigations of attentional bias. Gardner, Morrell, Watson, and Sandoval [23] analyzed attentional patterns in obese and normal-weight males and females while the participants were estimating their body size on a computer screen, and found that obese individuals focused longer on their waist region compared to controls. The authors did not assess the perceived attractiveness of the various body regions; nevertheless, the waist is a highly weight-relevant body site [24], which is closely related to negative body image and body dissatisfaction. The aforementioned study by Svaldi et al. [18] compared overweight women with and without BED. The authors found that both groups were more attentive to unattractive than to attractive body regions, and that this effect was more evident in the BED group and for the assessment of the subject’s own picture. In addition, they found preliminary evidence that BMI might play a role, but as BMI was a confounder, no clear conclusions could be drawn.

Although the majority of studies have only included females, three studies including men all reported that this attentional bias also applies to males: Gardner et al. [23] in a group of normal-weight and obese individuals, and Hewig et al. [25] and Cho and Lee [26] in a group of normal-weight students.

To summarize, findings from eye-movement studies on attentional bias towards body information are scarce. As the available studies differ in many respects, conclusions can only be drawn with caution. The aim of the present study was to examine overt selective visual attention and influences of body image-related variables in overweight individuals. We investigated (1) the overweight participants’ (OW) overt attention (gaze position) in comparison to a normal-weight group (NW) when confronted with a photo of their own body, (2) the specificity of gaze patterns by presenting a photo of a BMI- and gender-matched control person, and (3) gender-specific differences. In a more exploratory manner, we wanted to explore whether disturbed body image predicts attentional bias when viewing one’s own body. We hypothesized that compared to the NW group, the OW group would show (1) significantly longer fixations on their own unattractive body parts and shorter fixations on their own attractive body parts; and (2) significantly longer fixations on the attractive body parts of the control persons. With respect to gender differences, we assumed that the attentional bias applies to both genders, but is more pronounced in women. We further supposed that disturbed body image may contribute to explain this hypothesized attentional bias regarding one’s own body.

## Materials and Methods

### Participants

We recruited 28 normal-weight (16 females) and 30 overweight or obese participants (18 females) from the University of Potsdam and from an obesity support group. The overweight group consisted of 17 non-obese (12 females) and 13 obese participants (6 females). Sample characteristics for demographic and anthropometric data are summarized in Table 1. In the NW group, the mean age was lower and the proportion of students higher than in the OW group. When we controlled for the proportion of students, group differences in age were no longer significant (*F* (1, 55) = 1.07, *p* = .31). All participants showed normal or corrected-to-normal vision. The study was approved by the local ethics committee.

**Table 1:** Sample characteristics.

	NW		OW		test statistics		test statistics	
					group		gender	
	female	male	female	male				
	*n* = 16	*n* = 12	*n* = 18	*n* = 12	Χ^2^ (df) / *F*(df)	*p*	Χ^2^ (df) / *F*(df)	*p*
**students**								
(%)	93.75	91.67	61.11	83.33	Χ^2^ (1) = 3.87	.049	Χ^2^ (1) = 0.65	.422
**age**								
*M*	23.69	25.50	27.83	27.67				
(SD)	(2.70)	(2.84)	(7.81)	(9.09)				
range	19–29	21–30	20–45	20–51	*F*(1, 54) = 3.53	.066	*F*(1, 54) = 0.24	.626
**BMI**								
*M*	21.27	22.19	32.05	32.12				
(SD)	(1.55)	(1.52)	(6.06)	(6.06)				
range	18.60–24.70	19.80–24.90	26.90–47.90	25.00–44.25	*F*(1, 53) = 66.72	< .001	*F*(1, 53) = 0.07	.800
**waist circumference**								
*M*	74.04	79.98	101.16	108.37				
(SD)	(6.15)	(9.28)	(14.53)	(21.16)				
range	60–88	75–90	84–145	89–163	F(1, 53) = 55.96	< .001	F(1, 53) = 3.27	.076

### Procedure

All participants completed the following two sessions:

#### Session 1

First, participants completed a questionnaire to assess their body image and eating behavior. In addition, anthropometric data (weight, height, waist circumference) were collected. Following this, black-and-white photographs were taken under standardized conditions in the university’s photo studio. Each participant was photographed from 4 different angles (front, left, right, back) wearing plain underwear in front of a gray background. The compilation of the photographs also included the computer-assisted removal of identifiable marks (e.g., tattoos, piercings) and the blurring of the faces.

#### Session 2

The second session, which was scheduled on average 5.8 months after the first session, began with the eye-tracking procedure. Prior to the experiment, a test trial of four pictures, representing a gender-matched test picture from the four angles, was conducted. During the main trial, participants viewed their own set of pictures, followed by the BMI-and gender-matched control body pictures. As we had anonymized our stimulus material (e.g., blurring the head region), we considered it important to make participants aware that the first picture would show their own body. This aimed at minimizing orientation reactions and facilitating social comparison (see [27]) by using the subject’s own picture as the reference picture. In the next step, the participants viewed the pictures of the five remaining control sets. Pictures of all sets were presented in a sequence of the four cardinal angles, each for a duration of 10 seconds. After eye tracking was completed, the pictures (front and back view) were again presented on the screen, once again beginning with the subject’s own body and following the same sequence as during the eye tracking. This time, participants were asked to rate the attractiveness of the defined body regions for each set (front and back view).

Participants received a reimbursement of 30 € (20 € after the first session, 10 € following the second session).

### Materials

In line with international guidelines [28], all of the participants were assigned to one of the six different BMI categories (underweight: BMI < 18.5 kg/m^2^, lower normal weight: BMI 18.5–20.4 kg/m^2^, medium normal weight: BMI 20.5–22.4 kg/m^2^, higher normal weight: 22.5–24.9 kg/m^2^, overweight: 25.0–29.9 kg/m^2^, obese: ≥ 30 kg/m^2^). The stimulus set of control pictures was selected from the pool of photographs taken in session 1, based on highest representativeness for the respective BMI category.

### Eye-tracking apparatus

The material was presented on a 21'' CRT display (1280x1024, 100Hz) at a distance of 60 cm. Pictures subtended a visual angle of 21 by 28 degrees. Before each image presentation, a fixation check ensured that participants started their inspection in the navel region. When the fixation check failed, or following every fifth trial, the eye tracker was recalibrated with a nine-point grid. Eye movements were recorded monocularly by means of an SR EyeLink-1000 remote system (SR Research, Osgoode, Ontario, Canada) with a sampling rate of 1000 Hz. We conducted our experiment using the Psychophysics [29] and Eyelink Toolbox [30] extensions for MATLAB (The MathWorks Inc., Natick, MA, USA).

### Measures

#### Anthropometrics

Weight was measured with a digital scale (“Marsden MS-4102 L”) accurate to 100 grams, and height with a digital scale (“Soehnle 5003”) accurate to 1 centimeter. The sample was divided into two subgroups (NW: BMI 18.5–24.9 kg/m^2^; OW: BMI ≥ 25 kg/m^2^).

#### Disordered eating

The Eating Attitudes Test (EAT-26; [31]; German Translation: [32]) assesses symptoms of eating disorders. The EAT is a widely used self-report screening instrument and has proven to be reliable (Cronbach’s α = .90; [31]) and highly valid (*r* = .98 with EAT-40; [31]). The items were rated on a 6-point scale from “never” to “always”. We generated a sum score, with higher ratings indicating a higher level of eating pathology. In line with the literature [33], we used a cut-off score of 20 to identify persons with disordered eating.

#### Body dissatisfaction

Participants were asked to rate their satisfaction with their body (“How satisfied are you with your body?”) and with their weight (“How satisfied are you with your weight?”) on a 7-point Likert scale from “not at all satisfied” to “very satisfied”. We computed body dissatisfaction as a composite score of the two items (Cronbach’s α = .85), with higher scores indicating higher body dissatisfaction. This scale proved to be valid, showing significant correlations (*r* = .475, *p* = .019; controlling for age *r* = .510, *p* = .013) with the Contour Drawing Rating Scale [34] and the participants’ attractiveness ratings of their own body (*r* = -.489, *p* = .015; controlling for age *r* = -.531, *p* = .009).

#### Body checking behavior

The Body Checking Questionnaire (BCQ; [35]; German version: [36,37]) is a 23-item self-report inventory that assesses behaviors related to overall appearance, specific body parts and idiosyncratic body checking. Participants completed the questionnaire (e.g., “I check whether my thighs get wider when I sit down”) using a 5-point scale from “never” to “very often”, with higher scores indicating a higher frequency of the respective behavior (Cronbach’s α = .88; [37]). The total score showed an excellent internal consistency in our sample (Cronbach’s α = .92).

#### Avoidance behavior

The Body Image Avoidance Questionnaire (BIAQ; [38]; German version derived from [36]) was used to assess the avoidance of situations that provoke concerns about physical appearance (i.e. clothing, social activities, eating restraint). The BIAQ consists of 19 items (e.g., avoiding tight-fitting clothes or food-related situations) with answers provided on a 6-point scale (from “never” to “always”*)*. The total score showed an acceptable internal consistency in our sample (Cronbach’s α = .78).

#### Attractiveness rating

We divided each body, front and back, into 12 different regions of interest (ROIs), seven in the front view (arms, chest, cleavage, lower legs, stomach, thighs, waist) and five in the back view (arms, back, bottom, calves, thighs; cf. [36]). The participants had to rate the attractiveness of all ROIs in their own picture as well as in the control pictures on a 5-point Likert scale (“very unattractive” = -2, “unattractive = -1”, “neutral” = 0; “attractive = +1”, “very attractive” = +2). Scores higher than zero were coded as “attractive” and scores lower than zero as “unattractive”. For descriptive purposes, we transformed the scores on a 0–5 scale and calculated a sum score of the subjective attractiveness ratings of each picture seen by each participant, with higher values indicating higher perceived attractiveness.

### Data analysis

Saccades were detected in 2D velocity space (described in [39,40]) using thresholds for peak velocity (7 median-based standard deviations of the velocity distribution from the trial under scrutiny) and minimum duration (12ms). In accordance with common practice, first and last fixations (inter-saccade intervals) within trials and fixations of less than 50ms and more the 1000ms duration were excluded from analysis.

Data analysis was conducted using SPSS (version 21, IBM). The analyses of this study refer to the comparison of viewing times of one’s own body (OB) and the BMI- and gender-matched control body (CB). Since we did not assess the attractiveness ratings for the lateral view, we refer to the front and back view only. The three outcome variables were the percentage of fixation durations for attractive, neutral and unattractive ROIs based on the participants’ total viewing times (sum of front and back). Group differences in the three outcome variables were analyzed using two-factorial MANCOVA with the factors group (NW vs. OW) and gender. As the two groups differed in age, we included age as a covariate. For significant global effects in multivariate analysis, follow-up ANOVAs for each outcome will be reported. Attentional bias was calculated as the ratio of percentage of fixation durations on attractive to unattractive ROIs of one’s own body. Scores smaller than one indicate longer fixations on unattractive ROIs, i.e. a negative visual attention bias.

Predictors for attentional bias were analyzed by hierarchical logistic regression. For all statistical analyses, the alpha level was set at *p* < .05 (two-tailed); for group differences, information about effect sizes (η^2^) is included.

## Results

### Descriptive analyses

Results of descriptive analyses of body image-related variables and eating pathology are summarized in Table 2. In the OW group, five individuals showed elevated scores for disordered eating (EAT-26; χ^2^ (1) = 5.11, *p* = .024).

**Table 2:** Descriptive results for body image-related variables.

	NW		OW		test statistics		test statistics	
					group		gender	
	female	male	female	male				
	*n* = 16	*n* = 12	*n* = 18	*n* = 12	χ^2^ (df) / *F*(df)	*p*	χ^2^ (df) / *F*(df)	*p*
**disordered eating**								
(EAT-26>20; %)	0.00	0.00	22.22	8.33	χ^2^ (1) = 5.33	.021	χ^2^ (1) = 1.03	.310
**body dissatisfaction**								
*M*	32.81	34.03	75.00	63.19				
(SD)	(19.11)	(22.04)	(22.69)	(25.74)				
range	8.33–75.00	8.33–75.00	33.33–100	8.33–91.67	*F*(1,53) = 35.41	< .001	*F*(1, 53) = 0.70	.406
**attractiveness own body**								
*M*	58.17	65.28	31.94	49.13				
(SD)	(10.71)	(11.14)	(14.61)	(10.15)				
range	1.50–3.08	1.92–3.33	0.08–2.42	1.25–2.42	*F*(1,50) = 35.71	< .001	*F*(1,50) = 13.87	< .001
**attractiveness control body**								
*M*	63.39	61.98	39.81	36.11				
(SD)	(11.55)	(7.91)	(23.58)	(16.32)				
range	1.50–3.17	2.00–2.83	0.08–3.25	0.67–3.17	*F*(1,51) = 24.58	< .001	*F*(1,51) = 0.21	.647
**body checking**								
*M*	22.55	21.74	32.79	17.03				
(SD)	(9.82)	(7.70)	(16.61)	(20.87)				
range	10.87–42.39	6.52–31.52	9.78–63.04	2.17–81.52	*F*(1,53) = 1.21	.277	*F*(1,53) = 4.16	.046
**avoidance**								
*M*	26.32	25.11	40.49	30.92				
(SD)	(8.11)	(8.44)	(11.28)	(6.97)				
range	14.47–46.05	9.21–39.47	22.37–63.16	21.05–46.05	*F*(1,53) = 13.61	.001	*F* (1,53) = 5.39	.024

#### Body image

As expected, the OW and NW group differed in terms of components related to body image. Analyzing gender differences, women showed significantly increased body checking and avoidance, and lower ratings of their own body attractiveness. There was a significant gender x group interaction insofar as overweight women showed more body checking than normal-weight women, whereas no difference was observed between normal-weight and overweight men (*F* (1, 53) = 4.16, *p* = .046; see Table 2).

#### Attractiveness ratings

**Fig 1:**
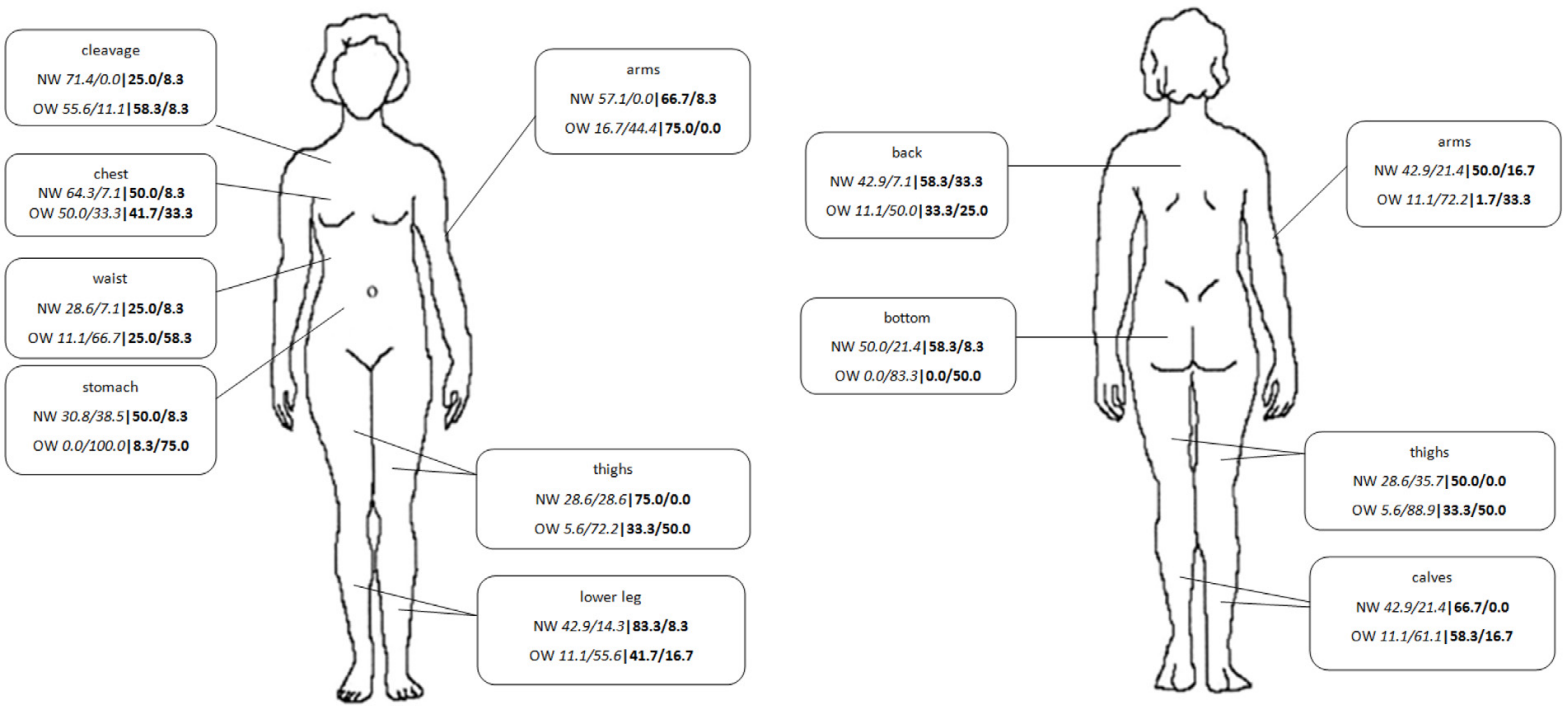
Results for the percentage of attractiveness ratings for all regions of interest (ROIs). Allocation on the basis of weight status (normal weight (NW) vs. overweight (OW)), gender (female: italics vs. male: bold) and attractiveness (attractive/ unattractive).

Both groups rated the cleavage region as their most attractive body region (OW: 55.6%; NW: 71.4%) and the stomach region as the least attractive (OW: 100.0%; NW: 38.5%). However, overweight and normal-weight men differed in their ratings of their most attractive and unattractive regions: Overweight men rated their arms in the front view as their most attractive region (75.0%) and their stomach as their least attractive region (75.0%), whereas normal-weight men judged their lower legs to be their most attractive body region (83.3%) and their back as their least attractive one (33.3%). These attractiveness ratings for each ROI (depicted in Fig 1) were later used as the basis for the eye-movement analysis.

When analyzing the number of body regions rated as attractive, unattractive, or neutral, we found highly significant group and gender differences. The OW group rated significantly more regions as unattractive. This applies both to the subject’s own body and the control body. Women from the OW group had the highest number of own unattractive ROIs (interaction effect *F* (1, 53) = 5.12, *p* = .028, η^2^ = .088). The results are provided in detail in the supplementary material online (S1 Table).

### Analyses of eye movements

Preliminary analyses with respect to the fixation duration on the bodies versus on the background did not show effects for group or gender either while looking at one’s own body (group *t* (56) = -1.26, *p* = .215; gender *t* (56) = -0.45, p = .656) or at the control body (group *t* (56) = -1.80, *p* = .078; gender *t* (56) = 0.68, *p* = .502).

#### Subject’s own body

**Fig 2:**
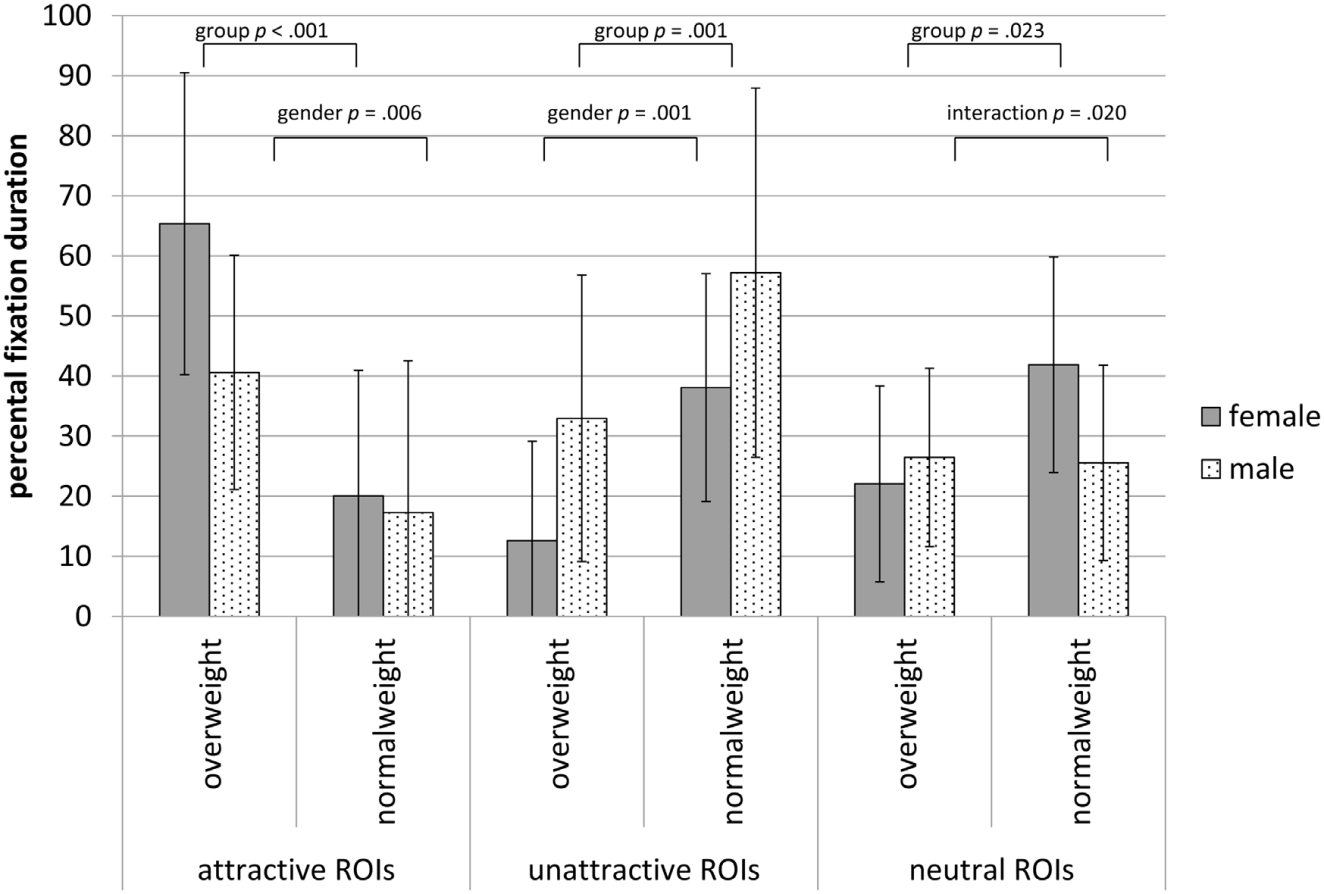
Percentage of dwell time for one’s own body (mean, standard deviation) for attractive, unattractive and neutral ROIs. Displayed are *p*-values of between-subjects analysis, following significant multivariate analysis (group *F* (2, 50) = 12.96; *p* < .001, η^2^ = .341; gender *F* (2, 50) = 5.77, *p* = .006, η^2^ = .187; interaction *F* (2, 50) = 3.11, *p* = .053, η^2^ = .111; covariate age *F* (2, 50) = 0.66, *p* = .422, η^2^ = .013).

The OW group focused significantly longer on attractive ROIs than the NW group (*F* (1, 51) = 26.41, *p* < .001, η^2^ = .341), which was contrary to our hypothesis. In addition, women directed their gaze significantly longer on attractive ROIs compared to men (*F* (1, 51) = 4.99, *p* = .030, η^2^ = .089). With respect to unattractive regions, we also found main effects for group and gender: Individuals from the NW group as well as men looked significantly longer at unattractive body regions (*F* (1, 51) = 13.47, *p* = .001, η^2^ = .209; resp. *F* (1, 51) = 11.59, *p* = .001, η^2^ = .185). Concerning neutral ROIs, the NW group showed significantly longer fixations (*F* (1, 51) = 5.52, *p* = .023, η^2^ = .098). We found an interaction effect with gender (*F* (1, 51) = 5.80, *p* = .020, η^2^ = .102), with women in the NW group showing the longest fixation duration (see also Fig 2).

#### BMI- and gender-matched control body

**Fig 3:**
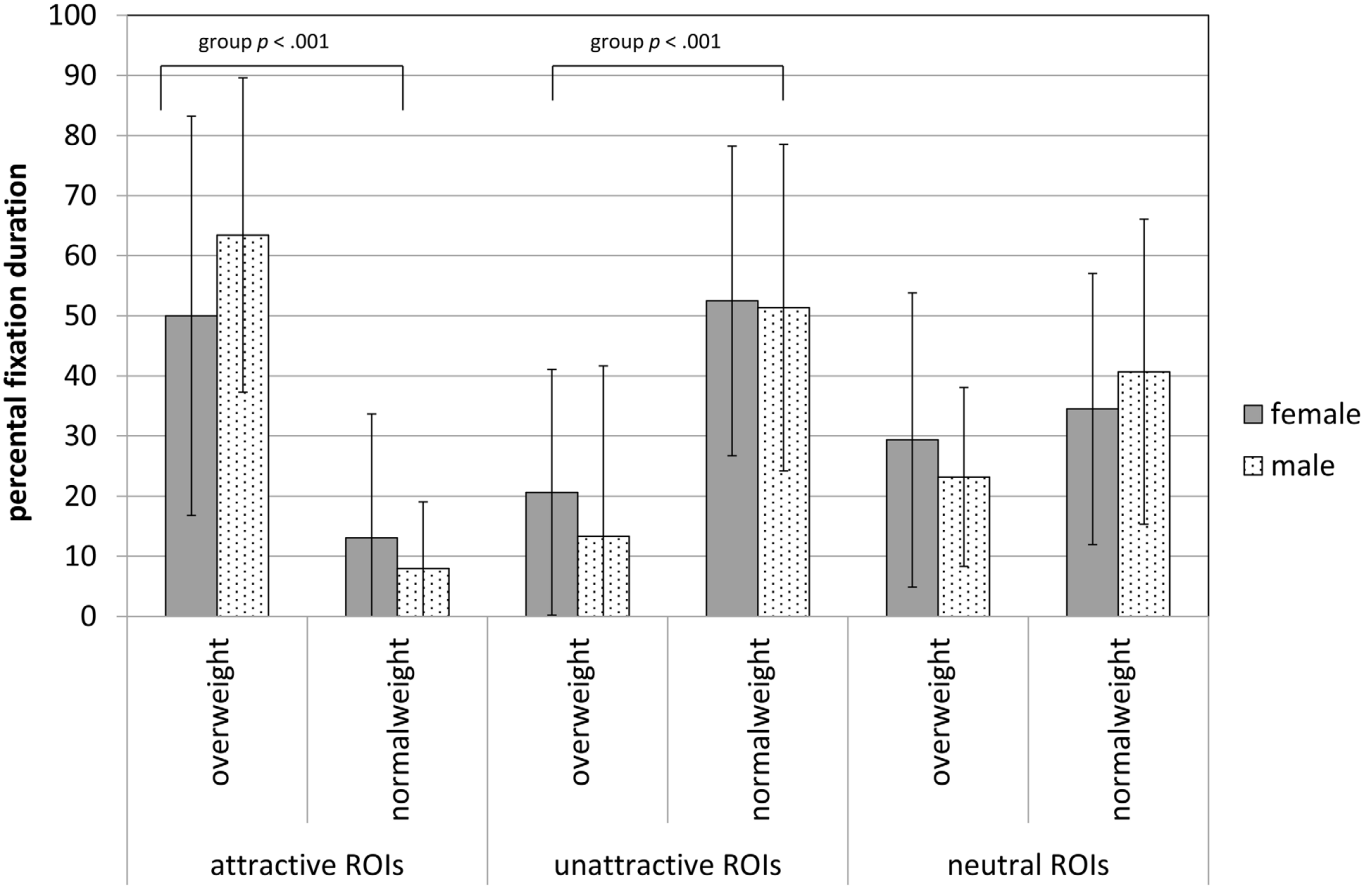
Percentage of dwell time for the control body (mean, standard deviation) for attractive, unattractive and neutral ROIs. Displayed are *p*-values of between-subjects analysis, following significant multivariate analysis (group *F* (2, 50) = 20.27, *p* < .001, η^2^ = .448), gender (*F* (2, 50) = 0.18, *p* = .837, η^2^ = .007), interaction (*F* 2, 50) = 1.21, *p* = .306, η^2^ = .046), covariate age *F* (2, 50) = 2.03, *p* = .142, η^2^ = .075).

The OW group focused significantly longer on attractive ROIs (*F* (1, 51) = 39.64, p < .001, η^2^ = .437; see Fig 3). Regarding the unattractive ROIs, the NW group showed significantly longer fixation durations (*F* (1, 51) = 22.33, *p* < .001, η^2^ = .304) than the OW group. There were no significant effects for neutral ROIs in the control body.

To assess the effects of recruitment strategy and disordered eating, we reran all analyses excluding persons with a high risk of eating disorders (OW: *n* = 5) and the members of the obesity support group (OW: *n* = 6), respectively. The main effects for weight status remained stable for one’s own body and also for the control body. The detailed information about these comparisons can be found in the supplementary material provided online (S2 Table).

### Attentional bias

Attentional bias was calculated as within-subject ratio of the fixation duration on attractive ROIs to unattractive ROIs. A positive bias means relatively longer fixations on attractive regions, and a negative bias means relatively longer fixations on unattractive regions. Our analyses of attentional biases revealed significant differences with respect to weight (*χ* ^2^ (1) = 6.53, *p* = .011; OW 34.6% negative bias; NW 73.1% negative bias), but not with respect to gender (*χ* ^2^ (1) = 1.44, *p* = .230; negative bias: women 48.0%, men 65.2%).

### Prediction of attentional bias

Due to the observed positive attentional bias in the OW group, we defined this variable as a dependent variable in a logistic regression analysis. In the first step, the variables of interest (according to our hypothesis) were tested for correlations with attentional bias on one’s own body. We found positive correlations with disturbed body image (body dissatisfaction *r* = .307, *p* = .036; body checking *r* = .484, *p* = .001; body image avoidance *r* = .418, *p* = .003,) as well as eating pathology *r* = .463, *p* = .001). For the logistic regression, we therefore entered age, gender and weight status (NW vs. OW) in the first step, and body dissatisfaction, eating pathology, body checking and body image avoidance in the second step. Results are summarized in Table 3. The final model was able to explain 61.5% of the variance in positive attentional bias. Weight status (normal weight as reference) and body checking significantly influenced the occurrence of a positive attentional bias by a decrease of 0.03 and increase of 1.23, respectively (weight status *b* = -3.568, *p* = .038, OR = 0.028, 95% CI [0.001, 0.825]; body checking *b* = 0.206, *p* = .017, OR = 1.229, 95% CI [1.037, 1.456]). Being of normal weight significantly reduces the occurrence of positive attentional bias, while body checking increases this positive bias. The other predictors did not significantly contribute to the observed positive bias

**Table 3:** Logistic regression for the prediction of a positive attentional bias.

	b	*p*	OR	95% CI	
				lower	upper
Step 1					
age	0.108	.235	1.114	0.932	1.330
gender (0 = male,1 = female)	-0.985	.143	0.373	0.100	1.394
group (0 = OW, 1 = NW)	-1.625	.015	0.197	0.053	0.725
Step 2					
age	.130	.267	1.139	.905	1.433
gender (0 = male,1 = female)	-.367	.673	0.693	0.126	3.818
group (0 = OW, 1 = NW)	-3.568	.038	0.028	.001	.825
body dissatisfaction	.022	.361	1.022	.975	1.072
disordered eating	.025	.755	1.025	.877	1.198
body checking	.206	.017	1.229	1.037	1.456
body image avoidance	.067	.316	1.069	.938	1.218

*Notes*. OW = overweight; NW = normal weight. For step 1, *R* ^2^ = .26 (Nagelkerke), χ^2^ (3) = 10.32, *p* = .016; for step 2 *R* ^2^ = .62 (Nagelkerke), χ^2^ (7) = 29.44, *p* < .001.

## Discussion

Despite the fact that overweight is associated with a high level of body dissatisfaction and behavioral manifestations of a disturbed body image, little is known about the visual attention patterns of overweight adults.

In line with the literature (e.g., [6,12,17,41,42]), descriptive analyses revealed that the overweight group exhibited lower overall attractiveness ratings and increased levels of body image avoidance, body dissatisfaction and eating pathology. Contrary to our expectations (e.g., [9,11,43]), we did not find significant differences in the amount of body checking between the overweight and the normal-weight adults. One possible explanation might be that the literature mainly focuses on homogenous overweight samples seeking treatment or displaying increased levels of eating pathology, whereas our sample was quite heterogeneous and included mainly non-clinical overweight adults. In accordance with previous studies (e.g., [42,44]), the women scored higher on body image avoidance and body checking. However, contrary to previous reports (e.g., [41,45]), males and females in our sample did not differ in their level of body dissatisfaction or disordered eating. One could argue that weight status is more crucial than gender in explaining body dissatisfaction and eating pathology. For instance, binge eating has been found to be nearly equally distributed among obese men and women (e.g., [46]).

In the eye-movement analyses, we found significant differences between the NW and OW group for body regions both of one’s own and the control body. Especially for the attractive body regions, effect sizes were large and revealed an unexpected pattern: The overweight adults looked longer at attractive body parts and shorter at unattractive ones compared to the normal-weight adults. These results are contrary to our hypotheses and to previous findings in various respects. First, the results in our overweight group are comparable to the pattern reported for normal-weight or body-satisfied people [17,47,48]. Second, the attentional bias towards attractive regions occurred despite the high values for body dissatisfaction, eating symptomatology and body image avoidance. These disturbed behavior patterns are claimed to be the consequence of a cognitive bias in favor of selectively processing weight-related information (such as paying attention to certain body regions; [49]). Hence, one would expect to find the same pattern in an overweight and obviously more strongly affected sample. Third, our overweight sample rated very few regions as attractive or neutral, while the opposite pattern was found in the normal-weight group. Despite this, the overweight sample showed longer fixation times on these fewer attractive regions. Our results contradict the reported negative attentional bias for overweight binge-eaters and non-binge-eaters [18] or the observation that a higher BMI is associated with a longer dwelling time on unattractive regions [48]. On the other hand, studies employing a dot-probe paradigm [50,51] have shown that individuals with a low or medium BMI are particularly susceptible to at least a more pronounced bias.

In contrast to the literature (e.g., [17,18,48]), we observed a quite stable pattern across the stimulus material presented, as fixations on attractive and unattractive body regions did not change when viewing a control body. When viewing one’s own body, certain internal viewing patterns (“schemas”) might have been activated and applied to the task [16,27,49]. When viewing the subsequent picture (control picture), results suggest that the same pattern of attention allocation might be applied and comparison processes might then have referred to the body regions with the same valence.

But how can these findings be integrated into current knowledge? It is claimed that an attention shift towards unattractive body regions is maladaptive, as it may foster a negative evaluation of one’s own body [49,52]. Our results might suggest that overweight persons exhibit a visual avoidance pattern in order to reduce their own discomfort (e.g., [53,54]). Seifert, Arnell, and Kiviniemi [55] observed that women scoring high on body dissatisfaction found obese bodies less salient and consequently paid less attention to them. Our overweight sample, due to high levels of body dissatisfaction, might have actively avoided looking at their unattractive regions by directing their attention to their attractive ones as a strategy to enhance and protect self-esteem [56]. The experience of discrimination and negative perceptions by others (which is common among overweight populations) is associated with a devaluation of physical attractiveness. Accordingly, the affirmation of positive characteristics might protect against the deterioration of one’s psychosocial well-being [57]. In line with this interpretation are the results reported by Smeets, Jansen, and Roefs [58], who successfully induced a positive attention bias leading to a decrease in body dissatisfaction. This might suggest that focusing on attractive regions provides overweight individuals with more valid information about their actual weight status than focusing on regions with a more pronounced fat accumulation.

In addition, we found first evidence for gender-specific patterns, but only for subjects’ own bodies. The female pattern matched the biases found in the overweight group, whereas men showed the same bias found in the normal-weight group. Irrespective of weight status, men tended to focus on the unattractive body regions—a pattern reported by Jansen et al. [17] for females with higher levels of disordered eating. To cope with the negative emotional feelings associated with the confrontation with their own physical flaws, they direct their gaze to their attractive body regions. Unfortunately, we did not ask our participants to verbalize their cognitions and emotions during the experiment. Research on the effects of body exposure found that eating-disordered women reported more negative cognitions and thoughts when looking at themselves in a mirror [59,60]. We did not find gender effects for the control body. This is in line with results by Hewig et al. [25], who analyzed attentional bias among women and men with an elevated drive for thinness in gender-matched control pictures. Taken together, our observed gender effect seems to mirror the results of the overall weight status and suggests that men and women react differently when confronted with their own pictures.

To gain a deeper insight into the factors involved in the attentional bias observed in our overweight group, we conducted a logistic regression analysis, which revealed that weight status (normal weight) and body checking influence the occurrence of a positive attentional bias. The positive association between body checking and positive attentional bias is in accordance with results from a previous study which analyzed the relationship between body checking and attentional bias towards body-related cues [61]. Body checking is a very common behavior—even among healthy samples—and frequent body checking is considered as an act of reassurance-seeking that can reduce anxiety and distress [41]. Our eye-tracking instructions allowed the free inspection of full-body photographs and therefore included a subtle form of body checking [61]. The strong fixation on the very few body regions which were rated as attractive might indicate a high level of reassurance-seeking and checking of these regions [62].

Several strengths of this study should be emphasized: First, by including male participants, we were able to analyze gender-specific differences. Second, since body image is a multifactorial concept, we included several measures to account for this. Third, our study is one of the first in overweight adults to include a normal-weight control group. Fourth, the BMI values and recruitment strategies in our groups were rather heterogeneous, which supports the external validity of our reported results. Fifth, we assessed predictor variables for the regression analysis and eye-tracking measures in separate sessions. This ensured that answering the body image-related questionnaire did not immediately influence attentional patterns. While these advantages are important, several limitations should be kept in mind: First of all, the sample size in the subgroups is relatively small, limiting the power for the group x gender interaction effects on the univariate level. Clinical significance of eye tracking results can be evaluated by measures of effect sizes, which were largely in the lower range. As statistical power in our analyses was only sufficient to detect medium to large effects, we are unable to draw definitive conclusions. Due to the cross-sectional design, causal conclusions about pathways of influence and interrelations of variables are not possible. Moreover, our design does not allow a direct comparison between attention allocation towards one’s own versus the control body, since we did not counterbalance the order of the stimuli presentation, which might influence attention allocation as well [63]. Additional potential confounding variables in the eye-tracking session (such as mood; [64]) were not controlled for.

### Clinical implications

Based on the results of this basic research trial, we can derive only first cautious implications for therapeutic approaches for obese persons. According to the cognitive-behavioral theory of eating disorders [49], eating-disordered individuals consistently affirm their own assumption that their body is ugly and fat by focusing on unattractive body regions. This, in turn, promotes a cascade of inadequate weight-related behaviors. The finding of the reversed pattern in our study is compatible with the view that attention allocation towards attractive body parts might function as an automatic emotion regulatory strategy [65]. This might prevent overweight and obese individuals from ending up in a vicious circle of deteriorating their already poor body image even more.

Since our data do not provide information about potential mechanisms underlying this attentional pattern, further experiments are needed to validate this assumption. Nonetheless, our questionnaire data confirm that obese persons suffer from a negative body image. The associated negative consequences are well-described in the literature and underscore the need for treatments to improve body satisfaction and body image. In this regard, first evidence suggests that trying to alter the automatic processes like selective attention is a promising approach [65,66]. Previous reports on the positive effects of guided mirror exposure in the treatment of overweight individuals (e.g., [59,67]) underscore the importance of body image therapy as an integral component of a comprehensive treatment approach. Based on our findings, the assessment of attractive body parts can be used for mindful perception of these parts and thereby enhance acceptance and decrease the negative affective states with respect to one’s own body [68,69].

Our results further emphasize that a differentiated understanding of overweight and obese adults’ subjective body image is needed. Unlike individuals suffering from eating disorders, overweight persons are quite accurate in perceiving their own body dimensions (e.g., [10]). A thorough exploration of body scheme content by targeting distinct body regions might provide relevant insight into the cognitive processes underlying individuals’ high level of body dissatisfaction. This might foster our understanding of whether the attentional focus on attractive body regions serves as an adaptive emotion regulation strategy, and in this way motivates the person’s self- esteem.

The research on body image disturbances in obesity and its treatment are still in their infancy. Prospective studies which help us to gain a deeper insight into the emergence of poor body image among obese people are required. It is also important to note that not all obese individuals suffer from their weight status. Therefore, future research should focus on the protective factors for a healthy body image. This would be fruitful for the development of successful interventions.

## Supporting Information

S1 Table Analysis of the number of regions of interest (ROIs) rated as attractive, neutral or unattractive in the own body and control body. (DOCX)

S2 Table Analysis excluding eating symptomatic individuals and members of the obesity support group. S2A. Results for percental fixation duration on attractive, unattractive and neutral regions of interest for the own body—individuals with elevated eating pathology (n = 5) and members of the obesity support group (n = 6) being excluded. S2B. Results for percental fixation duration on attractive, unattractive and neutral regions of interest for the control body—individuals with elevated eating pathology (n = 5) and members of the obesity support group (n = 6) being excluded.
(DOCX)

## Abbreviations

AN: anorexia nervosa
ANCOVA: Analysis of covariance
BCQ: body checking questionnaire
BED: binge eating disorder
BIAQ: body image avoidance questionnaire
BMI: body mass index
BN: bulimia nervosa
CB: control body
EAT: eating attitudes test
MANCOVA: multivariate analysis of covariance
NW: normal weight
OB: own body
OW: overweight
ROIs: region(s) of interest
WHO: World Health Organization

## References

[1] Hansson LM, Rasmussen F. Attitudes towards obesity in the Swedish general population: The role of one's own body size, weight satisfaction, and controllability beliefs about obesity. Body Image. 2014; 11: 43–50. 10.1016/j.bodyim.2013.10.004 24268600

[2] Hilbert A, Rief W, Braehler E. Stigmatizing attitudes toward obesity in a representative population-based sample. Obesity. 2008; 16: 1529–1534. 10.1038/oby.2008.263 18464749

[3] Menzel JE, Schaefer LM, Burke NL, Mayhew LL, Brannick MT, Thompson JK. Appearance-related teasing, body dissatisfaction, and disordered eating: A meta-analysis. Body Image. 2010; 7: 261–270. 10.1016/j.bodyim.2010.05.004 20655287

[4] Puhl RM, Heuer CA. The stigma of obesity: A review and update. Obesity. 2009; 17: 941–964. 10.1038/oby.2008.636 19165161

[5] Thompson JK. Assessment of body image In: Allison DB, editor. Handbook of assessment methods for eating behaviors and weight related problems. Measures, theory, and research. California: Sage Publications; 1995 pp. 119–144.

[6] Nissen NK, Holm L. Literature review: Perceptions and management of body size among normal weight and moderately overweight people. Obes Rev. 2015; 16: 150–160. 10.1111/obr.12231 25487846

[7] Schwartz MB, Brownell KD. Obesity and body image. Body Image. 2004; 1: 43–56. 10.1016/S1740-1445(03)00007-X 18089140

[8] Frederick DA, Peplau LA, Lever J. The swimsuit issue: Correlates of body image in a sample of 52,677 heterosexual adults. Body Image. 2006; 3: 413–419. 10.1016/j.bodyim.2006.08.002 18089245

[9] Latner JD. Body checking and avoidance among behavioral weight-loss participants. Body Image. 2008; 5: 91–98. 10.1016/j.bodyim.2007.08.001 18405867

[10] Legenbauer T, Vocks S, Betz S, Báguena Puigcerver MJ, Benecke A, Troje NF, et al Differences in the nature of body image disturbances between female obese individuals with versus without a comorbid binge eating disorder: An exploratory study including static and dynamic aspects of body image. Behav Modif. 2011; 35: 162–186. 10.1177/0145445510393478 21324945

[11] Reas DL, Grilo CM, Masheb RM, Wilson GT. Body checking and avoidance in overweight patients with binge eating disorder. Int J Eat Disord. 2005; 37: 342–346. 10.1002/eat.20092 15856496

[12] Tsai SA, Lv N, Xiao L, Ma J. Gender differences in weight-related attitudes and behaviors among overweight and obese adults in the United States. Am J Mens Health. 2015: 1–10. 10.1177/1557988314567223 25595019

[13] Brooks S, Prince A, Stahl D, Campbell IC, Treasure J. A systematic review and meta-analysis of cognitive bias to food stimuli in people with disordered eating behaviour. Clin Psychol Rev. 2011; 31: 37–51. 10.1016/j.cpr.2010.09.006 21130935

[14] Dobson KS, Dozois DJ. Attentional biases in eating disorders: A meta-analytic review of Stroop performance. Clin Psychol Rev. 2004; 23: 1001–1022. 10.1016/j.cpr.2003.09.004 14729421

[15] Freeman R, Touyz S, Sara G, Rennie C, Gordon E, Beumont P. In the eye of the beholder: Processing body shape information in anorexic and bulimic patients. Int J Eat Disord. 1991; 10: 709–714.

[16] Lykins AD, Ferris T, Graham CA. Body region dissatisfaction predicts attention to body regions on other women. Body Image. 2014; 11: 404–408. 10.1016/j.bodyim.2014.05.003 25047004

[17] Jansen A, Nederkoorn C, Mulkens S. Selective visual attention for ugly and beautiful body parts in eating disorders. Behav Res Ther. 2005; 43: 183–196. 10.1016/j.brat.2004.01.003 15629749

[18] Svaldi J, Caffier D, Tuschen-Caffier B. Attention to ugly body parts is increased in women with binge eating disorder. Psychother Psychosom. 2011; 80: 186–188. 10.1159/000317538 21389757

[19] Blechert J, Nickert T, Caffier D, Tuschen-Caffier B. Social comparison and its relation to body dissatisfaction in bulimia nervosa: Evidence from eye movements. Psychosom Med. 2009; 71: 907–912. 10.1097/PSY.0b013e3181b4434d 19661192

[20] Horndasch S, Kratz O, Holczinger A, Heinrich H, Hönig F, Nöth E, et al "Looks do matter"-Visual attentional biases in adolescent girls with eating disorders viewing body images. Psychiatry Res. 2012; 198: 321–323. 10.1016/j.psychres.2011.12.029 22417927

[21] von Wietersheim J, Kunzl F, Hoffmann H, Glaub J, Rottler E, Traue HC. Selective attention of patients with anorexia nervosa while looking at pictures of their own body and the bodies of others: An exploratory study. Psychosom Med. 2012; 74: 107–113. 10.1097/PSY.0b013e31823ba787 22210238

[22] Faulconbridge LF, Bechtel CF. Depression and disordered eating in the obese person. Curr Obes Rep. 2014; 3: 127–136. 10.1007/s13679-013-0080-9 24678445 PMC3963183

[23] Gardner RM, Morrell JA, Watson DN, Sandoval SL. Eye movements and body size judgments in the obese. Int J Eat Disord. 1990; 9: 537–544. 10.1002/1098-108X(199009)9:5<537::AID-EAT2260090509>3.0.CO;2-A

[24] Thompson JK. The (mis)measurement of body image: Ten strategies to improve assessment for applied and research purposes. Body Image. 2004; 1: 7–14. 10.1016/S1740-1445(03)00004-4 18089137

[25] Hewig J, Cooper S, Trippe RH, Hecht H, Straube T, Miltner WHR. Drive for thinness and attention toward specific body parts in a nonclinical sample. Psychosom Med. 2008; 70: 729–736. 10.1097/PSY.0b013e31817e41d3 18606732

[26] Cho A, Lee J. Body dissatisfaction levels and gender differences in attentional biases toward idealized bodies. Body Image. 2013; 10: 95–102. 10.1016/j.bodyim.2012.09.005 23122552

[27] Svaldi J, Caffier D, Tuschen-Caffier B. Automatic and intentional processing of body pictures in binge eating disorder. Psychother Psychosom. 2012; 81: 52–53. 10.1159/000329110 22123164

[28] World Health Organization [WHO]. Physical status: The use and interpretation of anthropometry Report of a WHO Expert Committee (WHO Technical Report Series 854). Geneva; 1995. 8594834

[29] Brainard DH. The Psychophysics Toolbox. Spat Vis. 1997; 10: 433–436. 10.1163/156856897X00357 9176952

[30] Cornelissen FW, Peters EM, Palmer J. The Eyelink Toolbox: Eye tracking with MATLAB and the Psychophysics Toolbox. Behav Res Methods Instrum Comput. 2002; 34: 613–617. 10.3758/BF03195489 12564564

[31] Garner DM, Olmsted MP, Bohr Y, Garfinkel PE. The Eating Attitudes Test: Psychometric features and clinical correlates. Psychol Med. 1982; 12: 871–878. 10.1017/S0033291700049163 6961471

[32] Meermann R, Vandereycken W. Therapie der Magersucht und Bulimia nervosa Ein klinischer Leitfaden für den Praktiker. Berlin: De Gruyter; 1987. German.

[33] Berger U, Wick K, Hölling H, Schlack R, Bormann B, Brix C, et al Screening riskanten Essverhaltens bei 12-jährigen Mädchen und Jungen: Psychometrischer Vergleich der deutschsprachigen Versionen von SCOFF und EAT-26. Psychother Psychosom Med Psychol. 2011; 61: 311–318. German. 10.1055/s-0031-1271786 21432746

[34] Thompson MA, Gray JJ. Development and validation of a new body-image assessment scale. J Pers Assess. 1995; 64: 258–269. 10.1207/s15327752jpa6402_6 7722852

[35] Reas DL, Whisenhunt BL, Netemeyer R, Williamson DA. Development of the Body Checking Questionnaire: A self-report measure of body checking behaviors. Int J Eat Disord. 2002; 31: 324–333. 10.1002/eat.10012 11920995

[36] Vocks S, Legenbauer T. Körperbildtherapie bei Anorexia und Bulimia nervosa Ein kognitiv-verhaltenstherapeutisches Behandlungsprogramm. 2nd ed Göttingen: Hogrefe; 2010. German.

[37] Vocks S, Moswald C, Legenbauer T. Psychometrische Überprüfung einer deutschsprachigen Fassung des Body Checking Questionnaire (BCQ). Z Klin Psychol Psychother. 2008; 37: 131–140. German. 10.1026/1616-3443.37.2.131

[38] Rosen JC, Srebnik D, Saltzberg E, Wendt S. Development of a Body Image Avoidance Questionnaire. Psychol Assess. 1991; 3: 32–37. 10.1037/1040-3590.3.1.32

[39] Engbert R, Kliegl R. Microsaccades uncover the orientation of covert attention. Vision Res. 2003; 43: 1035–1045. 10.1016/S0042-6989(03)00084-1 12676246

[40] Engbert R, Mergenthaler K. Microsaccades are triggered by low retinal image slip. Proc Natl Acad Sci USA. 2006; 103: 7192–7197. 10.1073/pnas.0509557103 16632611 PMC1459039

[41] Latner JD. Body weight and body image in adults In: Cash T, editor. Encyclopedia of body image and human appearance. Amsterdam: Elsevier; 2012 pp. 264–269.

[42] Walker DC, Murray AD. Body image behaviors: Checking, fixing and avoiding In: Cash T, editor. Encyclopedia of body image and human appearance. Amsterdam: Elsevier; 2012 pp. 166–172.

[43] Grilo CM, Reas DL, Brody ML, Burke-Martindale CH, Rothschild BS, Masheb RM. Body checking and avoidance and the core features of eating disorders among obese men and women seeking bariatric surgery. Behav Res Ther. 2005; 43: 629–637. 10.1016/j.brat.2004.05.003 15865917

[44] Meyer C, McPartlan L, Rawlinson A, Bunting J, Waller G. Body-related behaviours and cognitions: Relationship to eating psychopathology in non-clinical women and men. Behav Cogn Psychother. 2011; 39: 591–600. 10.1017/S1352465811000270 21878138

[45] Forrester-Knauss C, Zemp Stutz E. Gender differences in disord ered eating and weight dissatisfaction in Swiss adults: Which factors matter. BMC Public Health. 2012; 12: 1–9. 10.1186/1471-2458-12-809 22992241 PMC3503783

[46] Striegel-Moore RH, Franko DL. Epidemiology of binge eating disorder. Int J Eat Disord. 2003; 34: 19–29. 10.1002/eat.10202 12900983

[47] Janelle CM, Hausenblas HA, Ellis R, Coombes SA, Duley AR. The time course of attentional allocation while women high and low in body dissatisfaction view self and model physiques. Psychol Health. 2009; 24: 351–366. 10.1080/08870440701697367 20204998

[48] Roefs A, Jansen A, Moresi S, Willems P, van Grootel S, van der Borgh A. Looking good. BMI, attractiveness bias and visual attention. Appetite. 2008; 51: 552–555. 10.1016/j.appet.2008.04.008 18495295

[49] Williamson DA, Muller SL, Reas DL, Thaw JM. Cognitive bias in eating disorders: Implications for theory and treatment. Behav Modif. 1999; 23: 556–577. 10.1177/0145445599234003 10533440

[50] Gao X, Li X, Yang X, Wang Y, Jackson T, Chen H. I can’t stop looking at them: Interactive effects of body mass index and weight dissatisfaction on attention towards body shape photographs. Body Image. 2013; 10: 191–199. 10.1016/j.bodyim.2012.12.005 23352761

[51] Glauert R, Rhodes G, Fink B, Grammer K. Body dissatisfaction and attentional bias to thin bodies. Int J Eat Disord. 2010; 43: 42–49. 10.1002/eat.20663 19260041

[52] Shafran R, Fairburn CG, Robinson P, Lask B. Body checking and its avoidance in eating disorders. Int J Eat Disord. 2004; 35: 93–101. 10.1002/eat.10228 14705162

[53] Janelle CM, Hausenblas HA, Fallon EA, Gardner RE. A visual search examination of attentional biases among individuals with high and low drive for thinness. Eat Weight Disord. 2003; 8: 138–144. 10.1007/BF03325003 12880191

[54] Cisler JM, Koster EH. Mechanisms of attentional biases towards threat in anxiety disorders: An integrative review. Clin Psychol Rev. 2010; 30: 203–216. 10.1016/j.cpr.2009.11.003 20005616 PMC2814889

[55] Seifert AL, Arnell KM, Kiviniemi MT. The relation of body dissatisfaction to salience of particular body sizes. Eat Weight Disord. 2008; 13: e84–90. 10.1007/BF03327510 19169068 PMC2744589

[56] Crocker J, Major B. Social stigma and self-esteem: The self-protective properties of stigma. Psychol Rev. 1989; 96: 608–630. 10.1037/0033-295x.96.4.608

[57] Powell PA, Simpson J, Overton PG. Self-affirming trait kindness regulates disgust toward one's physical appearance. Body Image. 2015; 12: 98–107. 10.1016/j.bodyim.2014.10.006 25462888

[58] Smeets E, Jansen A, Roefs A. Bias for the (un)attractive self: On the role of attention in causing body (dis)satisfaction. Health Psychol. 2011; 30: 360–367. 10.1037/a0022095 21553980

[59] Hilbert A, Tuschen-Caffier B, Vögele C. Effects of prolonged and repeated body image exposure in binge-eating disorder. J Psychosom Res. 2002; 52: 137–144. 10.1016/S0022-3999(01)00314-2 11897232

[60] Vocks S, Legenbauer T, Wachter A, Wucherer M, Kosfelder J. What happens in the course of body exposure? Emotional, cognitive, and physiological reactions to mirror confrontation in eating disorders. J Psychosom Res. 2007; 62: 231–239. 10.1016/j.jpsychores.2006.08.007 17270582

[61] Smeets E, Tiggemann M, Kemps E, Mills JS, Hollitt S, Roefs A, et al Body checking induces an attentional bias for body-related cues. Int J Eat Disord. 2011; 44: 50–57. 10.1002/eat.20776 19950112

[62] Mountford V, Haase A, Waller G. Body checking in the eating disorders: Associations between cognitions and behaviors. Int J Eat Disord. 2006; 39: 708–715. 10.1002/eat.20279 16868998

[63] Mele S, Cazzato V, Urgesi C. The importance of perceptual experience in the esthetic appreciation of the body. PLoS ONE. 2013; 8: e81378 10.1371/journal.pone.0081378 24324689 PMC3852268

[64] Smith E, Rieger E. An investigation of the effect of body dissatisfaction on selective attention toward negative shape and weight-related information. Int J Eat Disord. 2010; 43: 358–364. 10.1002/eat.20703 19536880

[65] Wadlinger HA, Isaacowitz DM. Fixing our focus: Training attention to regulate emotion. Pers Soc Psychol Rev. 2011; 15: 75–102. 10.1177/1088868310365565 20435804 PMC2970710

[66] Martijn C, Sheeran P, Wesseldijk L, Merrick H, Webb T, Roefs A, et al Evaluative conditioning makes slim models less desirable as standards for comparison and increases body satisfaction. Health Psychol. 2013; 32 10.1037/a0028592 22612560

[67] Jansen A, Bollen D, Tuschen-Caffier B, Roefs A, Tanghe A, Braet C. Mirror exposure reduces body dissatisfaction and anxiety in obese adolescents: A pilot study. Appetite. 2008; 51: 214–217. 10.1016/j.appet.2008.01.011 18342397

[68] Forman EM, Butryn ML, Manasse SM, Bradley LE. Acceptance-based behavioral treatment for weight control: A review and future directions. Curr Opin Psychol. 2015; 2: 1–4. 10.1016/j.copsyc.2014.12.020 PMC4472472 26101783

[69] Olson KL, Emery CF. Mindfulness and weight loss: A systematic review. Psychosom Med. 2015; 77: 59–67. 10.1097/PSY.0000000000000127 25490697

