# Supplementary material for: Analysis of Attentional Bias towards Attractive and Unattractive Body Regions among Overweight Males and Females: An Eye-Movement Study
Source: PLoS One. 2015 Oct 19;10(10):e0140813. doi: 10.1371/journal.pone.0140813 (PMC4610678; doi:10.1371/journal.pone.0140813)
Supplement: S1 Table — (DOCX) [file pone.0140813.s001.docx]

Table S1. Analysis of the number of regions of interest (ROIs) rated as attractive, neutral or unattractive in the own body and control body

|  | **NW** | | **OW** | | **test statistic** | **test statistic** |
| --- | --- | --- | --- | --- | --- | --- |
|  | **female** | **male** | **female** | **male** | **group** | **gender** |
|  | *n* = 16 | *n* = 12 | *n* = 18 | *n* = 12 | *p* | *p* |
| attractive ROIs  *M*  (SD)  range | 4.62 / *5.43*  (2.92) / *(3.31)*  0-10 / *0-10* | 6.58 / *5.83*  (2.97) / *(2.37)*  2-10 / *1-9* | 1.89 / *2.94*  (2.05) / *(2.86)*  0-7 / *0-9* | 4.50 / *1.92*  (2.50) / *(3.26)*  0-7 / *0-11* | .004 / *.001^a^* | .001 / *.767^b^* |
| neutral ROIs  *M*  (SD)  range | 4.06 / *3.81*  (2.59) / *(2.61)*  0-8 / *0-10* | 4.33 / *5.25*  (2.35) / *(1.96)*  2-10 / *2-10* | 2.72 / *3.44*  (1.53) / *(1.75)*  0-8 / *1-5* | 3.33 / *3.50*  (2.19) / *(1.68)*  0-7 / *0-8* | .013 / *1.45*^c^ | .525 / *.234*^d^ |
| unattractive ROIs  *M*  (SD)  range | 1.75 / *1.25*  (1.84) / *(2.35)*  0-8 / *0-7* | 1.08 / *0.92*  (1.51) / *(1.16)*  0-3 / *0-4* | 7.39 / *5.61*  (2.77) / *(4.24)*  0-12 / *2-12* | 4.17 / *6.58*  (1.47) / *(3.03)*  0-11 / *2-7* | <.001 / *<.001*^e^ | .001 / *.808*^f^ |

Notes: Test statistics for the own body / *control body*

a *F* (1,53) = 9.08 / *F* *(1,53) = 12.71*; ƞ2 = .146/ *.193*

b *F* (1,53) = 6.65 / *F* *(1,53) = 2.19*; ƞ2 = .111/ *.040*

c *F*(1,53) = 56.43 / *F* *(1,53) = 31.55*; ƞ2 = .516/ .*373*

d *F* (1,53) = 11.84 / *F* *(1,53) = 0.09*; ƞ2= .183 /*.002*

e *F* (1,53) = 0.41 / *F* *(1,53) = 1.45*; ƞ2=.008 / *.027*

f *F* (1,53) = 12.43 / *F* *(1,53) = 0.06*; ƞ2=.190 / *.001*
