# Supplementary material for: Analysis of Attentional Bias towards Attractive and Unattractive Body Regions among Overweight Males and Females: An Eye-Movement Study
Source: PLoS One. 2015 Oct 19;10(10):e0140813. doi: 10.1371/journal.pone.0140813 (PMC4610678; doi:10.1371/journal.pone.0140813)
Supplement: S2 Table — S2A. Results for percental fixation duration on attractive, unattractive and neutral regions of interest for the own body—individuals with elevated eating pathology (n = 5) and members of the obesity support group (n = 6) being excluded. S2B. Results for percental fixation duration on attractive, unattractive and neutral regions of interest for the control body—individuals with elevated eating pathology (n = 5) and members of the obesity support group (n = 6) being excluded. (DOCX) [file pone.0140813.s002.docx]

**Supplement 2: Analysis excluding eating symptomatic individuals and members of the obesity support group**

Table S2a. *Results for percental fixation duration on attractive, unattractive and neutral regions of interest for the own body - individuals with elevated eating pathology (n = 5) and members of the obesity support group (n = 6) being excluded*

|  | **NW** | | **OW** | | **test statistics**  ***group*** | | | **test statistics**  ***gender*** | | |
| --- | --- | --- | --- | --- | --- | --- | --- | --- | --- | --- |
| **ROIs** | **female**  ***n*=14** | **male**  ***n*=12** | **female**  ***n*=10** | **male**  ***n*=10** | ***F*(df)** | ***p*** | **η²** | ***F*(df)** | ***p*** | **η²** |
| **attractive**  ***M***  **(SD)** | 20.04  (20.93) | 17.26  (25.29) | 58.71  (28.69) | 37.44  (19.95) | *F* (1,41) = 16.87 | < .001 | .292 | *F* (1,41) = 2.58 | .116 | .059 |
| **unattractive**  ***M***  **(SD)** | 38.07  (18.96) | 57.20  (30.74) | 15.95  (20.57) | 39.11  (21.00) | *F* (1,41) = 8.31 | .006 | .198 | *F* (1,41) = 9.87 | .003 | .194 |
| **neutral**  ***M***  **(SD)** | 41.89  (17.94) | 25.54  (16.24) | 25.34  (18.41) | 23.45  (14.45) | *F* (1,41) = 3.49 | .069 | .078 | *F* (1,41) = 4.33 | .044 | .095 |

Note. ROIs = regions of interest; for neutral ROIs significant gender*group interaction (*F* (1. 50) = 5.13. *p* = .028. η² = .093).

Table S2b. *Results for percental fixation duration on attractive, unattractive and neutral regions of interest for the control body - individuals with elevated eating pathology (n = 5) and members of the obesity support group (n = 6) being exclude*d

|  | **NW** | | **OW** | | **test statistics**  ***group*** | | | **test statistics**  ***gender*** | | |
| --- | --- | --- | --- | --- | --- | --- | --- | --- | --- | --- |
| **ROIs** | **female**  ***n*=14** | **male**  ***n*=12** | **female**  ***n*=10** | **male**  ***n*=10** | ***F*(df)** | ***p*** | **η²** | ***F*(df)** | ***p*** | **η²** |
| **attractive**  ***M***  **(SD)** | 13.04  (20.61) | 7.99  (11.06) | 32.32  (14.54) | 59.27  (26.71) | *F* (1,41) = 38.31 | <.001 | .483 | *F* (1,41) = 4.09 | .050 | .091 |
| **unattractive**  ***M***  **(SD)** | 52.46  (25.76) | 51.33  (27.17) | 28.31  (20.09) | 16.03  (30.52) | *F* (1,41) = 14.21 | .001 | .257 | *F* (1,41) = 0.62 | .434 | .015 |
| **neutral**  ***M***  **(SD)** | 34.50  (22.54) | 40.69  (25.37) | 39.37  (21.13) | 24.70  (15.73) | *F* (1,41) = 0.73 | .398 | .017 | *F* (1,41) = 0.66 | .422 | .016 |

*Note.* ROIs = regions of interest; for attractive ROIs, significant interaction group x gender (*F* (1, 41) = 8.018, *p* = .007, η² = .164).
